# Short-term Influences on Suspended Particulate Matter Distribution in the Northern Gulf of Mexico: Satellite and Model Observations

**DOI:** 10.3390/s8074249

**Published:** 2008-07-15

**Authors:** Eurico J. D'Sa, Dong S. Ko

**Affiliations:** 1 Louisiana State University, Department of Oceanography and Coastal Sciences, Coastal Studies Institute, LA 70803, USA; 2 Naval Research Laboratory, Stennis Space Center, MS, USA

**Keywords:** winds, circulation model, suspended particulate matter, northern Gulf of Mexico, Mississippi River, QuikSCAT, SeaWiFS

## Abstract

Energetic meteorological events such as frontal passages and hurricanes often impact coastal regions in the northern Gulf of Mexico that influence geochemical processes in the region. Satellite remote sensing data such as winds from QuikSCAT, suspended particulate matter (SPM) concentrations derived from SeaWiFS and the outputs (sea level and surface ocean currents) of a nested navy coastal ocean model (NCOM) were combined to assess the effects of frontal passages between 23-28 March 2005 on the physical properties and the SPM characteristics in the northern Gulf of Mexico. Typical changes in wind speed and direction associated with frontal passages were observed in the latest 12.5 km wind product from QuikSCAT with easterly winds before the frontal passage undergoing systematic shifts in direction and speed and turning northerly, northwesterly during a weak and a strong front on 23 and 27 March, respectively. A quantitative comparison of model sea level results with tide gauge observations suggest better correlations near the delta than in the western part of the Gulf with elevated sea levels along the coast before the frontal passage and a large drop in sea level following the frontal passage on 27 March. Model results of surface currents suggested strong response to wind forcing with westward and onshore currents before the frontal passage reversing into eastward, southeastward direction over a six day period from 23 to 28 March 2005. Surface SPM distribution derived from SeaWiFS ocean color data for two clear days on 23 and 28 March 2005 indicated SPM plumes to be oriented with the current field with increasing concentrations in nearshore waters due to resuspension and discharge from the rivers and bays and its seaward transport following the frontal passage. The backscattering spectral slope γ, a parameter sensitive to particle size distribution also indicated lower γ values (larger particles) in nearshore waters that decreased offshore (smaller particles). The use of both satellite and model results revealed the strong interactions between physical processes and the surface particulate field in response to the frontal passage in a large river-dominated coastal margin.

## 1. Introduction

The northern Gulf of Mexico is a region with large natural resources with its offshore oil and gas industry supplying approximately 20% of the U.S. energy demand and the fisheries industry one-third of its fisheries supply. It is influenced by the Mississippi-Atchafalaya River system, 7^th^ largest among the world's largest rivers in terms of water and sediment discharge that strongly influences the biogeochemical properties in the northern Gulf of Mexico. The Mississippi river discharges into the Gulf of Mexico through the birdfoot delta and through a secondary outlet to the west, the Atchafalaya river delta. The combined water and sediment discharge of the Mississippi and Atchafalaya rivers are about 530 × 109 m^3^ y^-1^ and 210 × 10^6^ tons yr^-1^ with 70% of the water and 50 to 60% of sediment discharged through the birdsfoot delta into deeper waters of the shelf while the rest discharges through the Atchafalaya River to the west onto a broad shallow shelf (Meade 1996). Coastal productivity has been shown to be strongly impacted by suspended sediments through its effect on light availability in waters influenced by the Mississippi river (Lohrenz et al. 1999). Between the months of October and April the region experiences frequent outbreaks of cold fronts or frontal passages that generally follow a pattern of changes in surface wind speed and direction, barometric pressure, temperature and humidity (Roberts et al. 1987; Mossa and Roberts 1990). These fronts are boundaries between streams of polar air moving southward and warm air flowing northward that impart energy to coastal and nearshore shelf environments (Roberts et al. 1987). It has been further suggested that these cold front passages numbering about 30-40 per year could have a larger cumulative impact along the coast and the continental shelf than the more energetic hurricanes through redistribution of sediments, transport of fluid mud, wave erosion, and other sedimentological and geomorphic changes to the coastal and nearshore shelf environment (Roberts et al. 1987; 2003). With approximately 80% of the US wetland loss having occurred in this region over the last half century (Stone and McBride 1998) improvements in monitoring the effects of frontal systems on the suspended particulate matter (SPM) (sediments and biogenic material) are important to support strategies for monitoring, response and mitigation in this coastal environment. In situ field measurements over large coastal regions are limited by costs and logistics while river-dominated systems exhibit strong spatial and temporal variations due to terrestrial inputs to the ocean. The use of satellite remote sensing data in combination with model results could be used to gain better insights on the physical processes and influences on SPM distribution in the coastal environment.

The large quantity of the suspended particulate load discharged by large rivers such as the Mississippi and Atchafalaya rivers and the uncertainties in the knowledge of their fluxes to the shelf and oceanic waters (Wollast 1991) suggests a need to understand their characteristics in these coastal environments. Remote sensing data from satellites such as the advanced very high resolution radiometer (AVHRR) and sea-viewing wide field sensor (SeaWiFS) have been useful in providing a synoptic view of the surface SPM distributions in the northern Gulf of Mexico (Walker and Hamack 2000; Myint and Walker 2002; Salisbury et al. 2004). In these studies, satellite estimates of SPM were obtained using single channel satellite reflectance bands of the AVHRR and the SeaWiFS sensors. Although the use of single reflectance bands in the visible have been shown to relate to SPM (Myint and Walker 2002; Miller and McKee 2004), the method is often sensitive to uncertainties related to atmospheric correction. In contrast errors are minimized in algorithms that use ratios of reflectance bands (e.g., R_rs_670/R_rs_555) that are close to near-infrared bands used for atmospheric correction (D'Sa et al. 2007). Further, the ratio algorithm has also been found useful to obtain estimates of the backscattering spectral slope γ, an optical parameter sensitive to particle size distribution (Loisel et al. 2006; D'Sa et al. 2007). Here we apply the ratio algorithms to SeaWiFS ocean color data to examine SPM dynamics (surface distribution and particle size characteristics) in combination with the outputs of a numerical model in the northern Gulf of Mexico during a frontal passage. Numerical models in conjunction with satellite data have been used to provide a better understanding of the processes in coastal and oceanic waters (Chassignet et al. 2005; Forget and André 2007; Mohn and White 2007). In the Gulf of Mexico, regional ocean circulation models such as the nested Navy Coastal Ocean Model (NCOM) (Martin, 2000; Ko et al. 2003) and satellite remote sensing have been used to gain a better understanding of the Loop Current and its eddy field which can strongly influence the circulation in the northern Gulf of Mexico (Chassignet et al. 2005). In this work we use the latest 12.5 km Level 2 wind product from QuikSCAT (Tang et al. 2004; Sharma and D'Sa 2008), the SeaWiFS derived SPM concentrations and the spectral backscattering slope parameter γ (Loisel et al. 2006; D'Sa et al. 2007) in combination with a nested high horizontal resolution (∼1.9 km) NCOM coastal model simulation of sea levels and surface currents to gain better insight on the suspended particle dynamics in a large river dominated coastal margin.

## 2. Methods and Data

### 2.1 Study site and data

**Figure 1.**
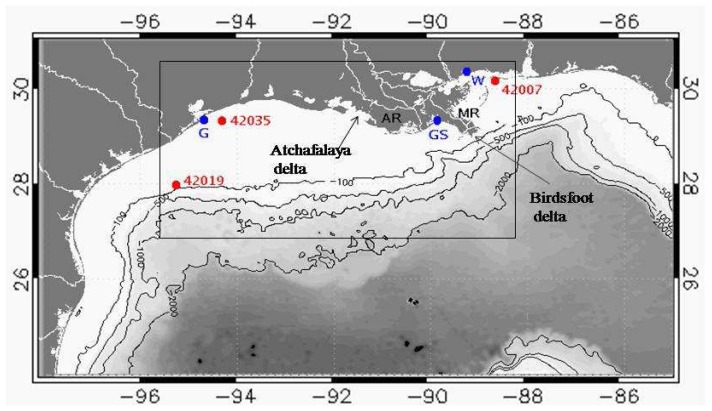
The study region in the northern Gulf of Mexico showing the domain (27-30.5°N latitude, 88.2-95.5°W longitude) of the nested coastal (MsLaTex) model and remote sensing data analysis (rectangular inset). The Mississippi River (MR) discharges through the birdsfoot delta while the Atchafalaya River (AR) lies to the west of MR. Smaller rivers and outlets include the Calcasieu, Sabine Lakes and the Trinity-San Jacinto estuary (G) located west of AR. The blue dots correspond to the location of three tide gauges located in Galveston (G)-Texas, Grand Isle (GS)-Louisiana and Waveland (W)-Mississippi. Also shown are the locations of three NOAA NDBC buoys within the study region.

The study area located in the Gulf of Mexico extends from 27-30.5° N latitude, 88.2-95.5° W longitude and includes coastal and oceanic waters off the coastal states of Mississippi, Louisiana and part of Texas (Figure 1, rectangular inset). Seasonal discharge from the Mississippi and Atchafalaya Rivers through the birdsfoot and Atchafalaya deltas results in a major region of freshwater influence along the coasts of Mississippi, Louisiana and Texas. Additional outflows from the bays and smaller rivers can also contribute to a lesser extent biogenic material and sediments to the inner shelf. High river discharge and the outbreak of cold fronts over the coastal region characterized the 23-28 March 2005 period considered in this study. Satellite data, model results and field data from tide gauges and NDBC buoys (significant wave height) in Figure 1 are considered in this study.

### 2.2 Remote sensing data

Satellite remote sensing data used in this study include winds from QuikSCAT and ocean color data from SeaWiFS during the frontal passage in March 2005 (Table 1). The Seawinds microwave scatterometer on QuikSCAT provides estimates of near-surface wind speed and direction over an 1800-km wide continuous swath. The latest QuikSCAT 12.5 km wind product (Tang et al. 2004) from Jet Propulsion Laboratory (JPL) (http://podaac-www.jpl.nasa.gov/) was downloaded and processed during the period of the frontal passage. This product was evaluated and found to be highly correlated to buoy measurements in the Gulf of Mexico (Sharma and D'Sa 2008). For ocean color, SeaWiFS Level L1 A data was downloaded from NASA DAAC and processed using NASA's SeaDAS 5.1 processing software using a regional SPM algorithm (D'Sa et al. 2007). SPM concentrations were found to be strongly correlated to atmospherically corrected SeaWiFS reflectance band ratio R_rs_670/R_rs_555 suggesting that the relationship could be used to derive surface SPM concentrations for the study region (D'Sa et al. 2007). The spectral slope γ of particulate backscattering which was found to be correlated to the reflectance ratio R_rs_670/R_rs_555 has been indicated to be sensitive to particle size distribution with a decrease in γ being attributed to increasing role of larger sized particles (Loisel et al. 2006). Thus SPM and γ (spectral backscattering slope) estimates were derived from SeaWiFS atmospherically corrected reflectance data (Gordon and Wang 1994) for two cloud free SeaWiFS imagery of 23 and 28 March using the empirical relationships given by (D'Sa et al. 2007)

(1) $$\text{SMP}=17.783\;{\left({\text{R}}_{\text{rs}}670/{\text{R}}_{\text{rs}}555\right)}^{1.11}$$

(2) $$\text{and},\gamma =3.46‐6.62\;\left({\text{R}}_{\text{rs}}670/{\text{R}}_{\text{rs}}555\right).$$

### 2.3 MsLaTex (Mississippi-Louisiana-Texas) coastal model

**Figure 2.**
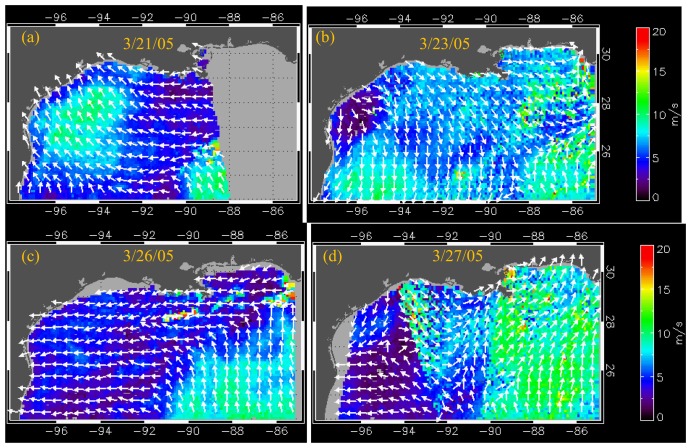
Sequence of QuikSCAT images for 21, 23, 26 and 28 March 2005 showing wind speed (image) and direction (vectors) during the frontal passage.

The Navy Coastal Ocean Model (NCOM) is a hybrid sigma-z vertical coordinate system based on the POM model that uses a nested modeling approach (Martin 2000; Ko et al. 2008). The high resolution Louisiana-Mississippi-Texas (MsLaTex) coastal model used in this study is nested within and receives boundary conditions from the regional 1/24° (∼6 km) horizontal resolution NCOM circulation model (Intra Americas Sea Nowcast/Forecast System or IASNFS) in operation for the Gulf of Mexico (e.g., Figure 2), the Caribbean, and parts of the western North Atlantic Ocean (Ko et al. 2003). The IASNFS regional model which assimilates real-time sea surface height anomaly data from three satellites (GFO, Jason-1 and ERS-2) and sea surface temperature data from AVHRR has been shown to produce realistic ocean circulation and sea level variations in the Gulf of Mexico and adjacent oceanic regions (Ko et al. 2003). The nested 372 × 200 grid MsLaTex model has 1/72° (∼ 1.9 km) horizontal resolution and vertical resolution of 19 layers on the shelf and 36 offshore. It spans the region between 27-30.5° N latitude and 88.2-95.5°W longitude (Figure 1, inset) that encompasses the Gulf coast states of Mississippi, Louisiana and Texas and includes shelf and oceanic waters in the northern Gulf of Mexico. Three hourly wind stresses, sea level air pressure and surface heat fluxes including solar radiation are applied for surface forcing from the Navy's Coupled Ocean/Atmosphere Mesoscale Prediction System (COAMPS) and the Fleet Numerical Meteorology Center (FNMOC). The model used the daily river discharge from the United States Geological Service (USGS) stations. The MsLaTex coastal ocean nowcast/forecast system is being developed to provide a 2-day forecast of sea-level, 3-d salinity, temperature and currents and has been run from 2002 with 3-hourly outputs of modeled results. In this study we examine selected model results on 23 and 28 March 2005 of sea level and surface currents of the study area during a frontal passage. Model derived sea level results are compared to tide gauge measurements at three tide gauge locations in Galveston (Texas), Grand Isle (Louisiana) and Waveland (Mississippi) (Figure 1) to assess the performance of model results with field data.

## 3. Results and discussion

### 3.1 Physical properties in the northern Gulf of Mexico

Discharge from the Mississippi-Atchafalaya river system, wind-induced coastal circulation, slope eddies and frontal systems are important physical influences on plume dynamics, optical properties, particle transport and mixing in the northern Gulf of Mexico (Nowlin and Parker 1974; Roberts et al. 1987; Walker et al. 1996; Wiseman et al. 1997; Salisbury et al. 2004; Walker et al. 2005; D'Sa and Miller 2003; D'Sa et al. 2006; 2007). Seasonal discharge from the Mississippi River with high discharge in the spring and low discharge in summer significantly influences the spatial extent of plume waters and the fluxes of freshwater, sediments, biogenic material and nutrients to the coastal waters. With tides in the northern Gulf of Mexico being diurnal and microtidal (tidal range less than 0.5 m), tidal currents are weak (Murray 1972). As such external forcing for the coastal currents are mainly wind stress and buoyancy flux from the rivers (Wiseman and Kelly 1994). Annual mean flow over the shelf is westward due to the persistent easterly winds over the northern Gulf of Mexico (Cochrane and Kelly 1986). However during the summer, westerly winds over the shelf cause a reversal of flow in the upcoast or easterly direction across the shelf (Nowlin et al. 2005). The seasonally shifting eastwards or westwards along-shelf surface currents generally tends to restrict river impacts to the continental shelf (Wiseman et al. 1997). On a seasonal scale, it has been shown that the spatial extent and orientation of the SPM distribution are significantly correlated to the wind field and the buoyancy of the river plumes (Salisbury et al. 2004).

During winter and early spring the northern Gulf of Mexico is strongly influenced by frontal passages that transfer energy to coastal waters and influence their physical and biogeochemical properties (Roberts et al. 1987; 2003; Moeller et al. 1993; Walker et al. 2000). Frontal passages play an important role in inducing large variations in sea level along the northern Gulf of Mexico. For instance, in the pre-frontal phase easterly, southeasterly winds cause coastal setup with a rising sea level followed by dropping water levels due to wind shifts in the frontal phase that can induce current reversals (Walker et al. 2005), marsh drainage and discharge of sediment-laden waters from bays onto the inner continental shelf (Mossa and Roberts 1990; Moeller et al. 1993). Resuspension of bottom sediments by strong winds during cold fronts, and the seaward-directed currents have been shown to transport suspended clays to the outer shelf (Roberts et al. 2003). Offshore, waters in the northern Gulf of Mexico are oligotrophic and often influenced by the eddy field associated with the presence of anticyclonic warm-core and cyclonic cold-core rings that are located over the continental slope and often impinge on the shelf (Nowlin et al. 2005).

### 3.2 Cold front passage – wind forcing effects on sea level and currents

The prevalent winds observed before the passage of a weak cold front on 23 March 2005 (Figure 2b) were predominantly easterly as indicated by the QuikSCAT wind field on 21 March 2005 (Figure 2a). Following the weak frontal passage, easterly winds again prevailed over the northern Gulf (Figure 2c) that was followed by the passage of a much stronger front on 27 March with winds speeds up to about 17 m s^-1^. Figure 2d provides a snapshot of the frontal system as it moved southeastward at an oblique orientation to the coast due to an eastward-migrating low-pressure system (Georgiou et al. 2005). Frontal passages are accompanied by a systematic shift in wind direction, atmospheric pressure and a large drop in air temperature (Roberts et al. 1987). In this study for the first time the latest 12.5 km QuikSCAT wind product has been used to provide a more synoptic view of the wind field associated with the frontal passages. Winds were strong and to the southwest reaching values of up to about 17 m s^-1^ at the boundary of the front. The front extended offshore for about 200 km from the coast being strongest nearshore and decreasing offshore. A similar pattern was observed in the COAMPS winds (not shown) that were applied for surface forcing of the MsLaTex coastal model.

Effects of such frontal passages have previously documented changes in water levels and increase in significant wave heights (Georgiou et al. 2005). At the NDBC buoy station 42035 near the Texas coast (Figure 1) significant wave height increased from 0.4 to 1.8 m following the peak wind speeds on 27 March that then relaxed within eight hours on 28 March to 5 m s^-1^ wind speed and 0.2 m significant wave height, respectively. Similarly at NDBC Buoy 42007 located along the Mississippi coast significant wave height increased to 1.43 m following the same frontal passage potentially contributing to resuspension in the region. Effects of the strong offshore winds have also been shown to result in seaward-directed currents that transport suspended sediments and river discharge to the outer shelf (Roberts et al. 2003). A notable feature observed by QuikSCAT imagery were the increased wind speeds over the warmer waters of the warm-core eddies located just below the delta and in the western Gulf (not shown).

**Figure 3.**
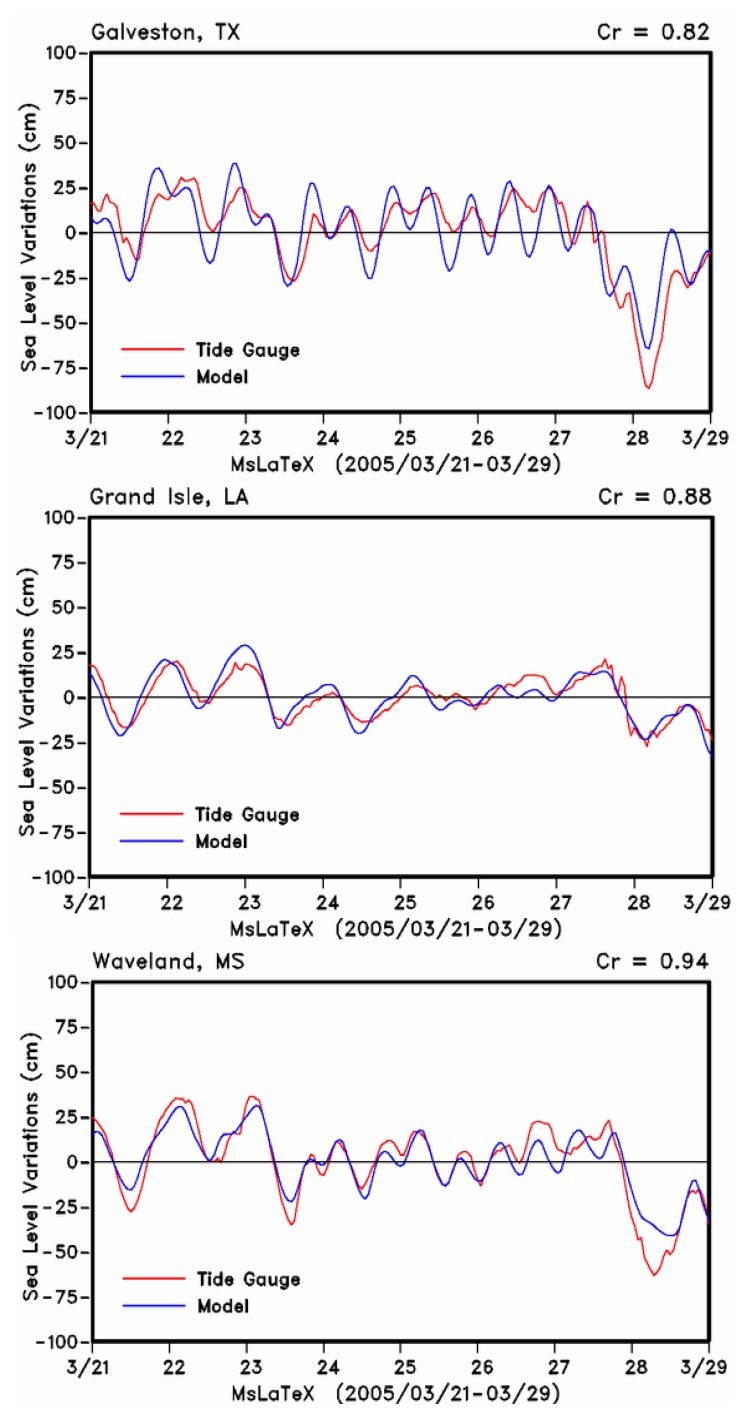
Sea level variations from model simulations (blue line) and tide gauge observations (red line) at (a) Galveston, Texas, (b) Grande Isle, Louisiana and (c) Waveland, Mississippi (shown in Fig. 1b) for the period 21-29 March 2005 corresponding to the frontal passages.

Wind data from weather stations in the northern Gulf of Mexico have been used to assess the impact of frontal passages in the northern Gulf of Mexico (Roberts et al. 1987; 2003; Walker et al. 2000; 2005). The QuikSCAT wind data (Figure 2) however provided a more synoptic view of the wind field and its potential effect on water levels along the Gulf coast. A comparison between modeled and tide gauge sea level data were made at three locations in the study area: Galveston-Texas on the western part of the Gulf, Grand Isle-Louisiana on the west side of the birdsfoot delta, and Waveland-Mississippi on the eastern side of the birdsfoot delta (Figures 1, 3). Overall, the comparisons indicated the model to realistically simulate sea levels with highest observed correlations near the delta at Waveland and Grand Isle tide gauge stations. Smallest variations in sea levels (± 0.25 m) were observed at the Grande Isle station which is immediately to the west of birdsfoot delta and therefore less impacted by the wind fetch. At this station tidal variations appear to be dominant. There was a good agreement between observed and simulated sea levels indicating that the model reproduced reasonably well the effects of the frontal system. Tide gauge measurements of sea level at Galveston and Waveland indicated higher water level setup along the western Gulf in the Texas coast and east of the birdsfoot delta along the Mississippi coast before the weak frontal passage of 23 March. Following the weak frontal passage, water levels dropped to about -0.30 m in Galveston and -0.4 m in Waveland and less than -0.15 m in Grand Isle (Figure 3). Subsequently easterly winds maintained more elevated sea levels (coastal setup) in the western Gulf than the eastern Gulf as indicated by the tide gauge measurements at the three stations. Following the more intense frontal passage of 27 March, tide gauge measurements indicated the largest drop in water levels of about 0.8 m at Galveston and 0.6 m in Waveland and the lowest drop of about 0.25 m at Grand Isle. However at both Galveston and Waveland model predictions were lower than those measured by the tide gauge station while they compared closely at the Grand Isle tide gauge station (Figure 3). These results suggest that the NCOM model still needs to be fine tuned during events such as frontal passages.

**Figure 4.**
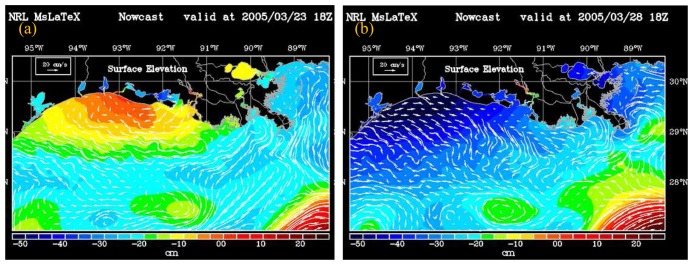
Model simulation of currents and surface elevation for 23^rd^ and 28^th^ March 2005. Current vectors are shown superimposed on the colored surface elevation map.

With model simulation of sea level comparing reasonably well with tide gauge measurements at three locations, model results of surface currents and elevations during the frontal passage on 23 and 27 (1800 hours UT) March 2005 should be well represented by the model predictions (Figure 4) which coincided with SeaWiFS satellite overpass through the study area. Although the model simulation on 23 March followed the weak frontal passage (Figure 2b), the surface elevation map suggested a small setup west of the Atchafalaya shelf. The weak frontal system did not influence the current field as westerly, southwesterly flows influenced by the easterly wind field still prevailed over the entire shelf in the northern Gulf of Mexico. However in the Atchafalaya shelf, weak easterly flows were simulated suggesting that the wind field may have influenced the flows in the shallow shelf waters. Flow speeds were variable with higher flows west of the birdsfoot delta suggesting enhanced buoyancy driven flows associated with the freshwater discharge from the Mississippi river. However, the eddy field comprising of anti-cyclonic warm core rings (sea level highs) and cyclonic cold-core rings (sea level lows) observed in the outer shelf and slope waters of the northern Gulf of Mexico (Figure 4a) appeared to influence the current field offshore. The strongest easterly current flows were observed to be associated with the anticyclonic warm-core ring south of the birdsfoot delta (Figure 4). East of birdsfoot delta, the projection of the delta into the Gulf interfered with the currents which flowed southwards and then southwesterly. Some of the flow however turned easterly under the influence of the same warm-core anticyclonic eddy (Figure 4a) potentially influencing the transport of particulate and dissolved organic matter offshore into the oligotrophic waters of the northern Gulf of Mexico.

Following the strong frontal passage of 27 March and the change in wind direction (Figure 2d) the model simulation of 28 March indicated a strong set down in surface elevation and an easterly, southeasterly reversal in the current flows due to the strong influence of wind forcing. Although model simulated surface elevations indicated a set down throughout the coast, the largest decrease in surface elevations were observed west of the Atchafalaya delta (Figure 4b). The large drop in surface elevation was also confirmed by the tide gauge measurements located at Galveston, Texas (Figure 1, Figure 3). A more complex current field was modeled offshore associated with the large anticyclonic warm-core eddy south of birdsfoot delta and two smaller eddies to the west. The model also indicated a westward drift of the large anti-cyclonic eddy, a common phenomenon observed in the northern Gulf of Mexico (Hamilton et al. 2002; Oey et al. 2005).

### 3.3 Frontal passage effects on particle dynamics

**Figure 5.**
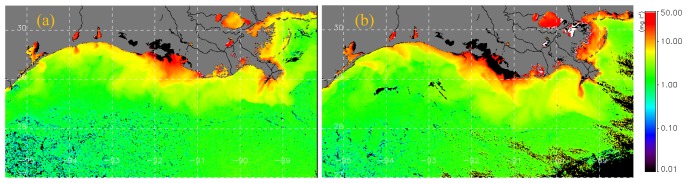
Suspended particulate matter (SPM) concentrations (mg l^-1^) derived from SeaWiFS data for the study region on (a) 23 March and (b) 28^th^ March 2005. Clouds are masked to black.

Plume dynamics and transport of dissolved and particulate material are strongly influenced by physical forcing such as circulation in the study region (Walker et al. 1996; Wiseman et al. 1997; Walker and Hammack 2000; Georgiou et al. 2005; D'Sa et al. 2007; Green et al. 2008). Modeled current flow field could thus provide an understanding on the redistribution of suspended particulate matter field (sediments and biogenic material) associated with the frontal passage in the northern Gulf of Mexico. SPM estimates derived from SeaWiFS ocean color data on 23 and 28 March (Figure 5) suggest strong linkages between the surface SPM distribution and the corresponding current field (Figure 4). A westward, southwestward oriented surface flow field simulated by the model was reflected in a similar orientation of the SPM in the nearshore and plume waters (Figure 5a). Highest estimates of SPM concentrations were observed in the shallow Atchafalaya delta and to the east around the birdsfoot delta associated with the outflows from the Atchafalaya and Mississippi rivers. Smaller plumes are also observed from outflows from the bays and smaller rivers and lakes located throughout the coast in the northern Gulf of Mexico. In the broad shallow Atchafalaya delta, higher SPM concentrations following the weak frontal passage of 23 March could be associated for example with resuspension of sediments or discharge from the Atchafalaya river (Roberts et al. 1987).

A change in the current flow field to a more easterly, southeasterly direction (Figure 4b) following the strong frontal passage of 27 March was reflected in the SPM plumes which appeared to be oriented similarly (5b). SPM plumes from the Atchafalaya and the Mississippi rivers, and smaller plumes from Calcasieu, Sabine rivers and the Trinity-San Jacinto estuary west of the Atchafalaya are also observed to be oriented in the southeastward direction (Figure 5b). The plumes appear elongated and oriented further offshore potentially from greater discharge of SPM from the rivers and bays suggesting seaward transport of SPM (Walker and Hamack 2000). An increase in SPM concentrations was also observed all along the nearshore coastal waters. East of the birdsfoot delta within the island chain (Breton and Chandeleur Sounds), higher SPM concentrations can be observed most probably due to a drop in sea level (Figure 4b) and resuspension in the shallow waters associated with the high wave heights observed in the region. South of the birdsfoot delta the outline of the warm-core eddy ring (Figure 4b) is weakly revealed by the low SPM concentrations. A water mass with higher SPM concentrations southwest of birdsfoot delta can be observed oriented in the southeasterly direction suggesting offshore transport under the influence of the counter-clockwise current field of the large warm core eddy. It has been suggested that the Louisiana-Texas slope eddies can provide mechanisms for material to be transported from the shelf to the deep basin and vice-versa (Hamilton et. al. 2002).

**Figure 6.**
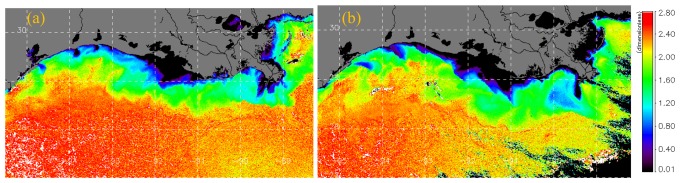
SeaWiFS derived imagery of spectral backscattering slope γ for (a) 23 March and (b) 28 March 2005.

Effects of the frontal passage on surface SPM dynamics were further examined using SeaWiFS ocean color derived estimates of the backscattering spectral slope γ (Figure 6) which provides an indication of the particle size distribution (Loisel et al. 2006). With low γ being associated with larger sized particles and increasing trend in γ with decrease in particle size, low γ (large particles) were observed nearshore and in the plume waters of the Atchafalaya and Mississippi rivers. Very low γ values are observed in the shallow waters of the Atchafalaya bay and close to the river discharge points suggesting larger particles in plume waters (Figure 6). With increasing distance offshore γ increases suggesting a decrease in particle size distribution. However, based on γ distribution in the satellite imagery, three regions can be discerned – a nearshore region with very low γ strongly influenced by river plume waters, resuspension and discharge from the various bays and smaller rivers, an offshore region with high values of γ (low particle size distribution) and an intermediate region lying between the more oligotrophic waters and the nearshore coastal waters comprising of particles intermediate between the offshore and nearshore waters. The orientation of the larger sized particles nearshore also suggests the strong influence of the southwestward current flows as indicated by the model simulation (Figure 4a) obtained at the same time as the satellite overpasss (Figure 6). A distinct front in particle size distribution is observed between the intermediate (γ between 1.2 and 2, green region) and the more offshore region (γ greater than 2, red region). The intermediate region is inshore of the 100-m isobath and may correspond to the wind-influenced shallow shelf region off the Louisiana-Texas coast wherein a significant positive wind stress-SPM correlation was observed aligned with the 50-m isobath (Salisbury et al. 2004). In shelf waters influenced by both the Atchafalaya and Mississippi rivers the particle front extends further offshore than the region west of the Atchafalaya delta with reduced river influences. The pattern however suggests that the particle front may indicate a region of freshwater influence along the shelf (Wiseman et al. 1997) that could also be influenced by enhanced surface concentrations of particulate matter such as phytoplankton.

Following the frontal passage of 27 March, the nearshore low γ distribution pattern changed to the southwesterly direction, appeared aligned with the flow field and its greater areal extent suggested the potential for the seaward transport of the larger sized particles from the various rivers and bays along the Lousiana-Texas coast (Figure 6b). In the shallow waters east of the birdsfoot delta values of γ decreased suggesting an increase in particle size distribution most probably due to resuspension related to wave activity following the frontal passage. In the offshore region we observed a general decrease in γ suggesting a small increase in particle size distribution that could potentially be due to greater mixing in the surface waters following the frontal passage.

## 4. Conclusions

The northern Gulf of Mexico receives large amounts of suspended particulate matter (sediments and biogenic material) from the largest river system in North America that along with energetic meteorological events such as frontal passages strongly impacts the biogeochemical processes in the region (Roberts et al. 1987; Lohrenz et al. 1999; Walker et al. 2000; 2005). The latest 12.5 km QuikSCAT data provided a good representation of the wind field during a frontal passage in March 2005 that in combination with selected outputs of a high resolution numerical circulation model and SPM estimates from SeaWiFS ocean color data allowed a better understanding of the SPM dynamics in the region. Model results of sea levels compared well with tide gauge measurements, however small differences were observed between tidal locations in Texas, Mississippi and Louisiana, with the best comparisons obtained at Grand Isle tide station located just west of the birdsfoot delta. Easterly, southeasterly winds observed before and after the passage of a weak frontal system on 23 March resulted in slightly elevated sea levels and westward currents along the coast. Following a strong frontal passage on 27 March, model results indicated a reversal in the coastal currents to eastward, southeastward direction. An examination of the SPM field indicated the orientation of the current flows strongly influenced the SPM distribution and its seaward transport which appeared enhanced following the frontal passage. In addition, the frontal system influenced the backscattering spectral slope γ, a parameter shown to be sensitive to the suspended particle size distribution. SeaWiFS estimates of γ suggested an increase in nearshore particle size in plume and nearshore waters probably due to increased discharge, mixing and resuspension associated with the frontal passage. In the offshore region model results of sea levels indicated the presence of an eddy field comprising of a large anticyclonic warm-core eddy below the birdsfoot delta that from the current and SPM imagery suggest an offshore transport of particulate matter. This study demonstrated that the combined use of satellite and model results provided a better understanding of the physical processes and its influence on the SPM dynamics in a large river-influenced coastal margin.

## Figures and Tables

**Table 1.** Dates in March 2005 (indicated by an ‘x’) when the QuikSCAT and SeaWiFS data were acquired and processed for the study area.

Satellite sensor/dates	3/21/05	3/23/05	3/26/05	3/27/05	3/28/05
QuikSCAT	x	x	x	x	
SeaWiFS		x			x
